# Associations of circulating matrix metalloproteinases and tissue inhibitors of matrix metalloproteinases with clinically relevant outcomes in idiopathic pulmonary fibrosis: Data from the IPF-PRO Registry

**DOI:** 10.1371/journal.pone.0312044

**Published:** 2024-10-17

**Authors:** Olawale Amubieya, Jamie L. Todd, Megan L. Neely, Robert J. Kaner, Joseph A. Lasky, Andrew Namen, Christian Hesslinger, Scott M. Palmer, S. Samuel Weigt, John A. Belperio

**Affiliations:** 1 Division of Pulmonary and Critical Care Medicine, David Geffen School of Medicine at UCLA, Los Angeles, California, United States of America; 2 Duke Clinical Research Institute, Durham, North Carolina, United States of America; 3 Department of Medicine, Duke University Medical Center, Durham, North Carolina, United States of America; 4 Department of Biostatistics and Bioinformatics, Duke University Medical Center, Durham, North Carolina, United States of America; 5 Departments of Medicine and Genetic Medicine, Weill Cornell Medicine, New York, New York, United States of America; 6 School of Medicine, Tulane University, New Orleans, Louisiana, United States of America; 7 Department of Internal Medicine, Section of Pulmonary, Critical Care and Allergy and Immunologic Diseases, Atrium Health Wake Forest School of Medicine, Winston-Salem, North Carolina, United States of America; 8 Translational Medicine and Clinical Pharmacology, Boehringer Ingelheim Pharma GmbH & Co. KG, Biberach, Germany; University of Vermont College of Medicine, UNITED STATES OF AMERICA

## Abstract

**Introduction:**

We assessed the prognostic utility of circulating levels of matrix metalloproteinases (MMPs) and tissue inhibitors of matrix metalloproteinases (TIMPs) in patients with idiopathic pulmonary fibrosis (IPF) in the IPF-PRO Registry.

**Methods:**

MMP and TIMP concentrations were quantified by ELISA in plasma from 300 patients. A Cox proportional hazard regression model was used to assess associations between select MMPs and TIMPs and death and disease progression (absolute decline in forced vital capacity ≥10% predicted, death, or lung transplant).

**Results:**

Over a median follow-up of 30.4 months, 98 patients died and 182 patients had disease progression. In unadjusted analyses, higher concentrations of MMPs 2, 3, 8 and 9 and TIMPs 1, 2 and 4 were associated with an increased risk of death. MMPs 2 and 8 and TIMP1 remained associated with death after adjustment for clinical factors. In unadjusted analyses, higher concentrations of MMPs 8 and 9 and TIMPs 1 and 4 were associated with an increased risk of disease progression. MMPs 8 and 9 and TIMP1 remained associated with progression after adjustment for clinical factors.

**Conclusion:**

Circulating levels of MMP8 and TIMP1 may provide information on the risk of outcomes in patients with IPF not captured by clinical measures.

## Introduction

Idiopathic pulmonary fibrosis (IPF) is a progressive fibrosing interstitial lung disease that primarily affects individuals over the age of 50 and has a poor prognosis [1, 2]. Two antifibrotic medications, pirfenidone and nintedanib, have been approved for the treatment of IPF, having been shown in clinical trials to slow disease progression [3, 4].

The pathogenesis of IPF involves excess deposition of extracellular matrix (ECM) proteins in the lung and dysregulated matrix remodeling, replacing normal lung architecture with fibrosis. Potential mechanisms include aberrant repair after pulmonary epithelial cell injury with fibroblast activation, epithelial to mesenchymal transition (EMT), collagen deposition, and immune cell dysfunction [5]. Matrix metalloproteinases (MMPs) are a family of zinc-dependent endopeptidases that are in part responsible for the turnover and degradation of ECM substrates. They are also involved in processes related to immunity and repair that include leukocyte activation, cell migration, and antimicrobial defense, as well as growth factor and chemokine processing [6]. Some MMPs are antifibrotic while others are pro-fibrotic. MMPs and their physiologic inhibitors, the tissue inhibitors of MMPs (TIMPs), are tightly regulated in the lung and play a role in the resolution of fibrosis as well as ECM deposition [6]. In keeping with their roles in the pathobiology of IPF, the utility of MMPs and TIMPs as indicators of disease behavior or prognosis in patients with IPF is an area of active investigation.

Early studies evaluating MMPs or TIMPs as predictors of clinical outcomes in patients with IPF focused on measuring these analytes in bronchoalveolar lavage fluid (BALF). This work, primarily conducted in small cohorts, suggested that MMPs 3, 8, 9, and 10 may be associated with disease progression [7, 8]. Measuring MMPs and TIMPs in the peripheral blood is of particular interest given the ease of testing. The MMP that has been most widely reported as of potential prognostic significance is MMP 7. A retrospective analysis of data from the INSPIRE study cohort found that circulating MMP 7, alongside the *MUC5B* polymorphism *rs35705950*, was associated with worse survival [9]. Other studies have reported associations between elevated MMP 7 and worse outcomes based on faster decline in lung function, worse overall survival, or worse transplant-free survival [10–14], although one study looking at 57 patients did not find higher serum or BALF MMP-7 levels to be associated with clinical deterioration or mortality [15]. A systematic review combining individual patient data from 1664 subjects across 9 studies found blood MMP 7 levels to be associated with increased mortality and faster disease progression [16].

The IPF-PRO Registry is a prospective multicenter registry of patients with IPF [17]. Previous analyses have shown that circulating MMP and TIMP levels at enrollment were broadly elevated in patients in this registry compared to a cohort of similar age and sex distribution without known lung disease, with MMP 8, MMP 9, and TIMP 1 being the top candidates to distinguish patients with IPF from controls [18]. In the current analyses, we used data from the IPF-PRO Registry to understand the prognostic utility of circulating levels of MMPs 1, 2, 3, 7, 8, 9, 12, and 13 and TIMPs 1, 2, and 4.

## Materials and methods

### Study cohort

The cohort consisted of 300 patients enrolled in the IPF-PRO Registry (NCT01915511) between June 2014 and February 2017 [17]. Patients with IPF that was diagnosed or confirmed at the enrolling center within the past 6 months were eligible for enrollment. This analysis included patients who had enrollment blood samples and data on critical clinical variables at enrollment (age, sex, height, smoking status, definite/probable/possible IPF according to the 2011 ATS/ERS/JRS/ALAT diagnostic criteria [19], FVC, DL_CO_, FEV_1_). Outcomes were ascertained from enrollment through June 2019.

The IPF-PRO Registry study obtained ethics approval at the data coordinating center (Duke Clinical Research Institute, Duke Institutional Review Board Protocol Number Pro00046131) and at every enrolling center (listed in the Acknowledgments). Additionally, ethics approval was granted by the Duke Institutional Review Board Protocol Number Pro00082241 to use the biosamples obtained as part of the IPF-PRO Registry for the analyses contained herein. All participants gave written informed consent.

### MMP and TIMP quantification

MMPs 1, 2, 3, 7, 8, 9, 12, 13 and TIMPs 1, 2, and 4 were quantified in plasma collected at enrollment using multiplexed Luminex immunoassays or standard ELISA kits. Samples that fell below the standard curve for MMP 1 (n = 23), MMP 8 (n = 48), MMP 12 (n = 7), or MMP 13 (n = 10) were extrapolated if feasible or assigned a concentration of half of the minimum observed value. No samples fell below the standard curve for MMPs 2, 3, 7, or 9 or TIMPs 1, 2, or 4.

### Statistical analyses

Data management and statistical analyses were completed using SAS 9.4 or R 3.4.1. As the distribution of some of the analytes had a strong right skew, the measured values for each analyte were log_2_ transformed to bring the distributions close to normal distributions and so stabilize the model fits. Time to death and to the composite of first occurrence of an absolute decline in FVC ≥10% predicted, death, or lung transplant were chosen as endpoints. The Kaplan-Meier method was used to obtain estimates of the cumulative event probability as a function of time and produce survival curves. A Cox proportional hazard regression model for time-to-first event was used to assess the unadjusted and adjusted associations between each circulating MMP or TIMP and each outcome. Adjustment variables included age, sex, DL_CO_ % predicted, FVC % predicted, supplemental oxygen use at rest or with activity, and antifibrotic medication use, all assessed at enrollment. Linearity and proportional hazards assumptions were assessed using the unadjusted model. No linearity violations were found. For analytes for which the proportional hazards assumption failed, an interaction term with time was included in the model as a time-dependent covariate and hazard ratios with 95% confidence intervals at 6, 12, and 24 months are presented to describe how the association changed during follow-up. The proportional hazards adjustments identified from the unadjusted models were applied to the adjusted models. For each outcome, P-values were corrected for multiple comparisons using the Benjamini-Hochberg procedure to control the false discovery rate (FDR) at 5%. The associations between clinical outcomes and MMP/TIMP ratios of interest based on established biological relationships were explored using similar methods to those described above (see S1 Methods).

The data required to replicate these analyses are available in the Duke University digital repository: https://doi.org/10.7924/r4ff40891.

## Results

### Baseline characteristics and outcomes

**Fig 1:**
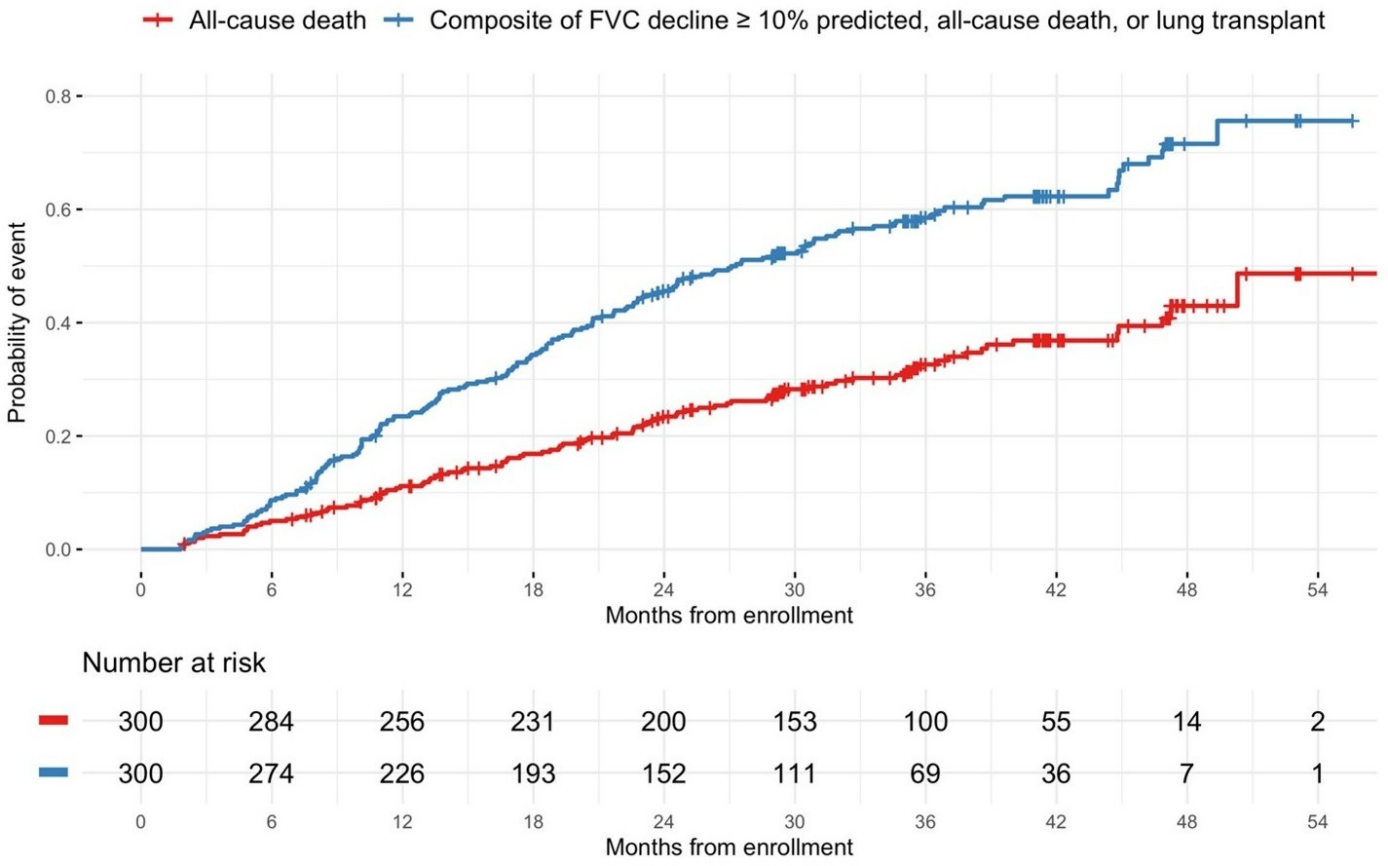
Time from enrollment to death (red) and to the composite of an absolute decline in forced vital capacity (FVC) ≥10% predicted, death, or lung transplant (blue).

The baseline characteristics of this cohort (n = 300) have been published [18]. Briefly, median (Q1, Q3) age was 70 (65, 75) years, 74% were men, 94% were white, and 67% were former smokers, 54% were taking nintedanib or pirfenidone, 20% were receiving supplemental oxygen at rest. Median (Q1, Q3) FVC % predicted was 69.7 (61.0, 80.2) and DL_CO_ % predicted was 40.5 (31.6, 49.4). Over a median (Q1, Q3) follow-up of 30.4 (20.1, 41.1) months, 98 patients died and 182 patients met the composite outcome of an absolute decline in FVC ≥10% predicted, death, or lung transplant. Among these patients, the first event was FVC decline for n = 110, death for n = 61, and lung transplant for n = 11. Kaplan-Meier curves for death and the composite of absolute decline in FVC ≥10% predicted, death, or lung transplant are shown in Fig 1.

### Associations between circulating MMPs and TIMPs and death

**Fig 2:**
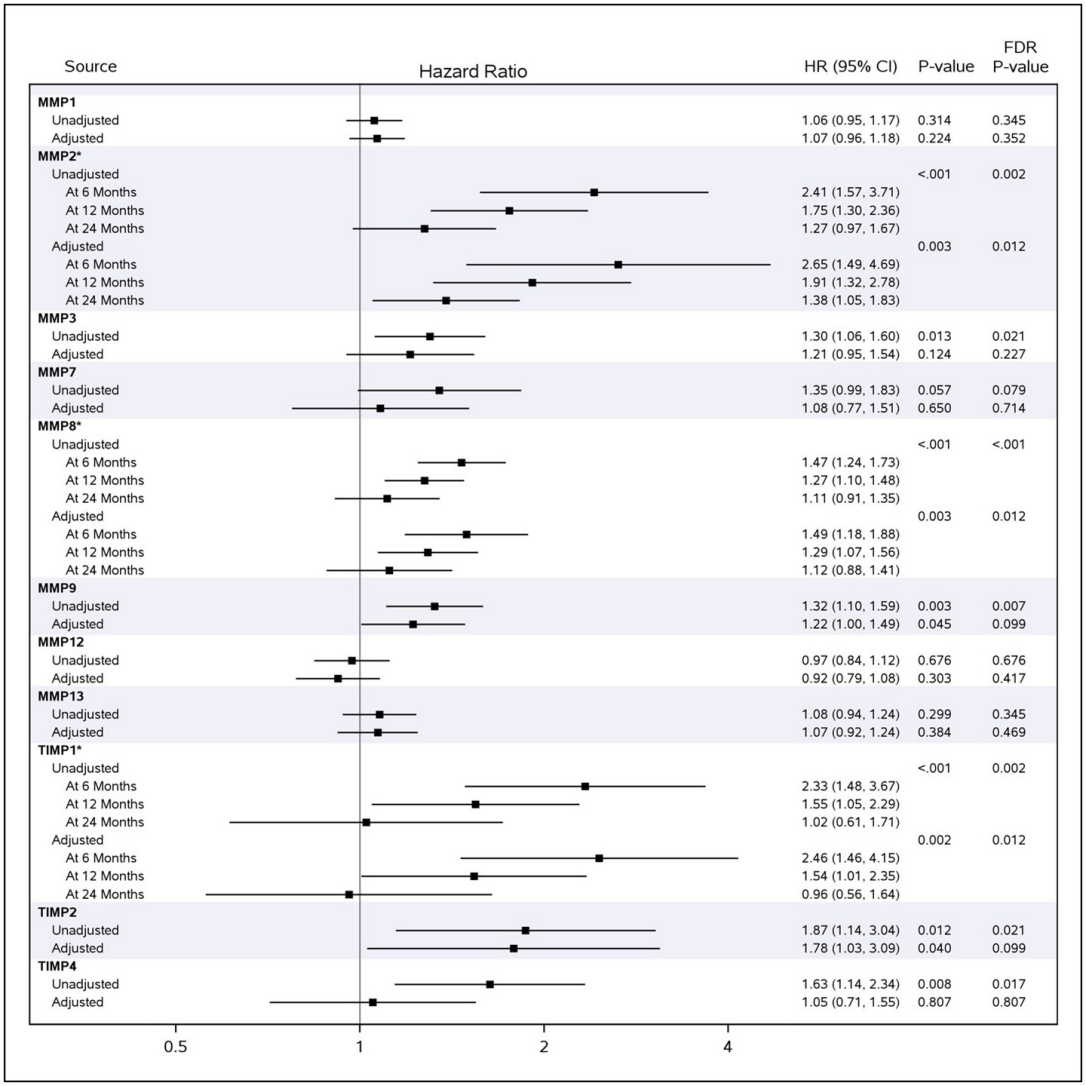
Associations between circulating MMPs and TIMPs at baseline and death. HRs per unit increase in baseline log2 (concentration) of each MMP or TIMP are shown. *Analyte violated proportional hazards assumption in Cox model. HR, hazard ratio; MMPs, matrix metalloproteinases; TIMPs, tissue inhibitors of MMPs.

In unadjusted analyses, higher concentrations of MMPs 2, 3, 8, and 9 and TIMPs 1, 2, and 4 were associated with an increased risk of death (Fig 2). MMP 2, MMP 8, and TIMP 1 remained significantly associated with death after adjustment for demographic and clinical factors (Fig 2). MMP 2, MMP 8, and TIMP 1 violated the proportional hazards assumption. For each of these analytes, the strength of the association was highest at 6 months and attenuated at later time-points, perhaps in part due to there being fewer patients at risk at the later time points. At 6 months, the unadjusted HRs (95% CI) per unit increase in baseline log_2_ (concentration) of MMP 2, MMP 8 and TIMP 1 were 2.41 (1.57, 3.71), 1.47 (1.24, 1.73) and 2.33 (1.48, 3.67), respectively. The adjusted 6-month HRs for MMP 2, MMP 8, and TIMP 1 were 2.65 (1.49, 4.69), 1.49 (1.18, 1.88) and 2.46 (1.46, 4.15), respectively. While we observed a trend toward an increased risk of death in patients with higher levels of MMP 7 in unadjusted analyses (unadjusted HR: 1.35; 95% CI 0.99, 1.83; p = 0.079), this association was attenuated after accounting for clinical variables (adjusted HR 1.08; 95% CI 0.77, 1.51, p = 0.714).

### Associations between circulating MMPs and TIMPs and composite of absolute decline in FVC ≥10% predicted, death, or lung transplant

**Fig 3:**
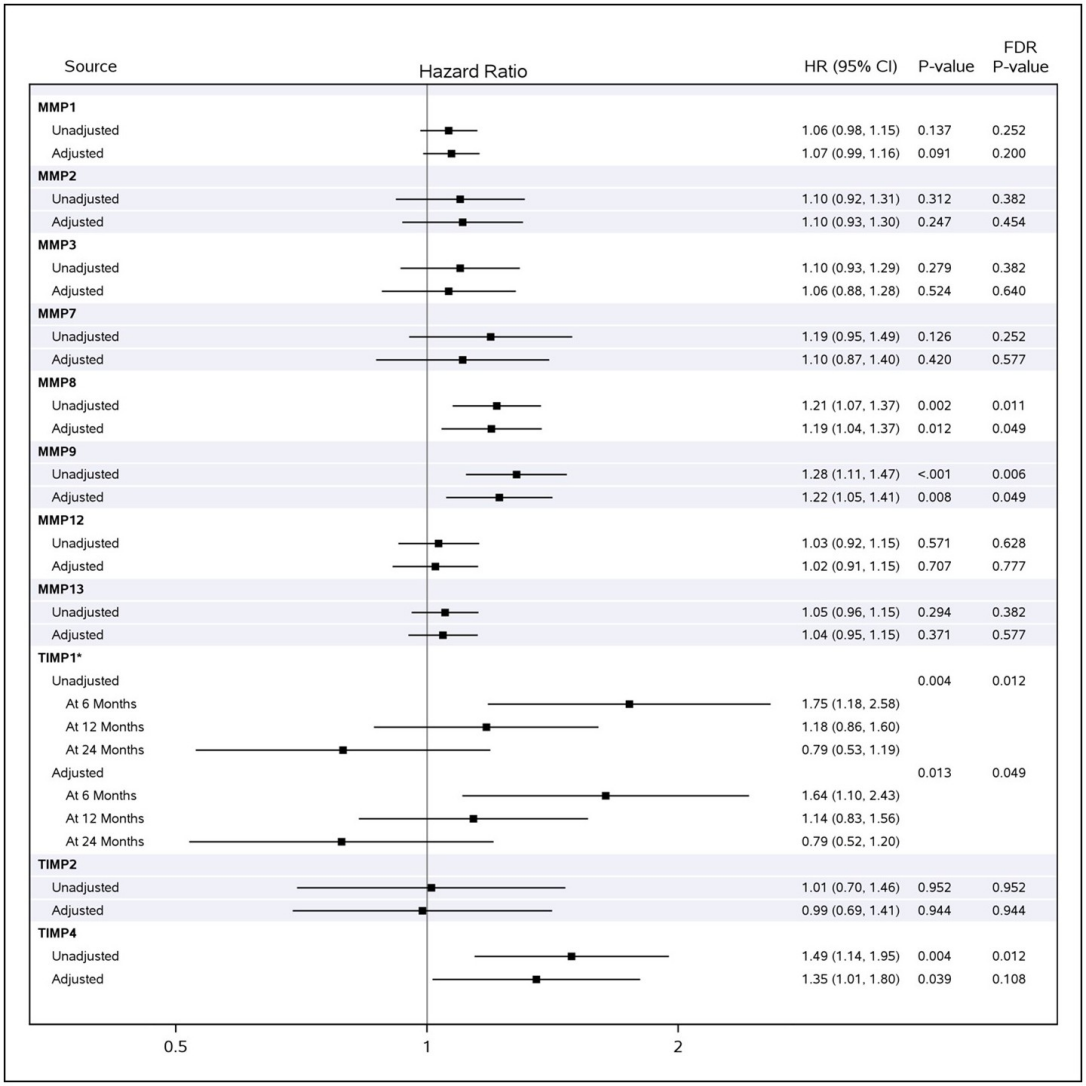
Associations between circulating MMPs and TIMPs at baseline and the composite outcome (absolute decline in forced vital capacity ≥10% predicted, death, or lung transplant). HRs per unit increase in baseline log2 (concentration) of each MMP or TIMP are shown. *Analyte violated proportional hazards assumption in Cox model. HR, hazard ratio; MMPs, matrix metalloproteinases; TIMPs, tissue inhibitors of MMPs.

In unadjusted analyses, higher concentrations of MMPs 8 and 9 and TIMPs 1 and 4 were associated with the composite outcome (Fig 3). MMPs 8 and 9 and TIMP 1 remained significantly associated with the composite outcome after adjustment for demographic and clinical factors (Fig 3). The unadjusted HR per unit increase in log_2_ (concentration) MMP 8 was 1.21 (1.07, 1.37) while the adjusted HR was 1.19 (1.04, 1.37). The unadjusted and adjusted HRs for MMP 9 were 1.28 (1.11, 1.47) and 1.22 (1.05, 1.41), respectively. TIMP 1 violated the proportional hazards assumption. The unadjusted 6-month HR for TIMP 1 was 1.75 (1.18, 2.58) and the adjusted HR was 1.64 (1.10, 2.43). Time-dependent receiver operating characteristic (ROC) curves for select MMPs at 6 months and 12 months post-enrollment are included in S1 Fig. Consideration of select MMP/TIMP ratios did not provide additional information about the risk of either clinical outcome beyond that conferred by the MMP or TIMP alone (see S2 and S3 Figs).

## Discussion

This study is among the first to quantify a wide array of circulating MMPs and TIMPs in a multicenter prospective cohort of well-characterized patients with IPF and to relate concentrations of MMPs and TIMPs to the risk of clinically relevant outcomes. Prior analyses of data from the IPF-PRO Registry elucidated the ability of individual and combinations of MMPs and TIMPs to discriminate between patients with IPF and controls without lung disease, with MMP 8, MMP 9, and TIMP 1 identified as the top candidates, and to identify patients with IPF with worse lung function [18]. The current study provides insights into how circulating levels of MMPs and TIMPs relate to the risk of disease progression and mortality. Higher plasma concentrations of MMP-2, MMP 8 and TIMP 1 were independently associated with mortality after controlling for clinical factors. Higher concentrations of MMPs 8 and 9 and TIMP 1 were associated with the composite of an absolute decline in FVC ≥10% predicted, death, or lung transplant after controlling for clinical factors. This suggests that MMP 8 and TIMP 1 may provide information on the risk of long-term outcomes in this patient population that is not captured by routine clinical measures.

The mechanisms underlying the activity of MMPs and TIMPs and the progression of IPF are incompletely understood. MMP 8 (collagenase 2) is expressed by peripheral blood monocytes, alveolar macrophages, bronchiolar epithelial cells (BECs), alveolar epithelial cells (AECs), and fibrocytes [20, 21]. MMP 8 levels are increased in bronchoalveolar lavage fluid and lung homogenates from patients with IPF [8, 20]. Elevated MMP 8 levels are found in the lungs of mice with bleomycin-induced fibrosis [22], and MMP 8 knockout mice demonstrate reduced lung fibrosis after bleomycin exposure [23]. These experimental data are consistent with our finding that higher MMP 8 concentrations in the peripheral blood were associated with worse outcomes. MMP-8 has also been implicated in the pathogenesis of pulmonary hypertension (PH), with studies demonstrating elevated levels in the pulmonary arteries and peripheral circulation of patients with PH, increased expression in rodent models of PH, and an association between elevated MMP 8 and right ventricular end diastolic volume in patients with PH [24, 25]. The relationship between MMP 8 and PH may have contributed to the observed association between higher levels of MMP 8 and worse outcomes in our study.

To our knowledge, the IPF-PRO Registry is the first large multicenter study in which circulating TIMPs have been measured in patients with IPF. We found that TIMP 1 concentrations conferred information about the risk of disease progression and death, even after accounting for clinical factors. TIMP 1 has been found in macrophages, fibroblast-like cells, and ECM and exhibits broad MMP inhibition. Levels of TIMP 1 are elevated in the sputum of patients with IPF [26]. TIMP 1 forms a complex with proMMP-9, preventing its activation by stromelysin [27]. Imbalance between TIMP 1 and MMPs impairs re-epithelialization after lung injury, potentially leading to aberrant repair [28]. TIMP 1 levels are elevated early after bleomycin administration in mice and appear to modulate the gelatinase activity of MMPs 2 and 9 [29]. Knockout of TIMP 1 in mice has not been shown to inhibit fibrosis in response to bleomycin, but has been associated with acute lung injury [30]. Together, these data suggest that increasing levels of TIMP 1 may be a physiologic mechanism to limit profibrotic MMPs, with a failure to do so associated with poor outcomes.

Neovascularization has been known to be present within areas of pulmonary fibrosis for many years [31]. Studies have demonstrated an imbalance in the levels of angiogenic chemokines (ELR^+^ CXC chemokines [CXCL5 and CXCL8]) and angiostatic chemokines (interferon-inducible ELR^-^ CXC chemokines [CXCL9, CXCL10, CXCL11]) in animal models of pulmonary fibrosis and lung tissue from patients with IPF [32]. Multiple studies have demonstrated associations between MMPs and TIMPs and angiogenesis, particularly MMP 2 and MMP 9 [33–36]. MMP 2 (gelatinase A) is expressed by BECs, AECs, fibroblasts, and fibrocytes [21, 37], and MMP-9 (gelatinase B) is expressed by AECs, neutrophils, alveolar macrophages, fibrocytes, and fibroblasts [38]. MMP 2 and MMP 9 have been shown to potentiate the pro-angiogenic chemokines involved in vascular remodeling in patients with IPF (CXCL5 and CXCL8) [39–42]. Studies in mice and tissue from patients with IPF demonstrate that increased CXCL5 and CXCL8 as compared to CXCL9 and CXCL10 leads to angiogenesis [35, 43]. Collectively, these studies suggest that MMP 2 and MMP 9 are altering chemokine biology that is pro-angiogenic, which is needed to support the fibroplasia involved in IPF.

Interestingly, in contrast to previous reports [9–14, 16], we did not find a significant association between circulating MMP 7 and clinical outcomes. There are several differences between our study and earlier research that may contribute to this discrepancy. The prior studies were conducted before the wide availability of antifibrotic therapy. One of the older studies involved patients who were taking immunosuppressive therapies, which may have altered MMP 7 levels [9]. The majority of the previous studies had shorter follow-up times than our study. In our analyses, MMP and TIMP concentrations were treated as continuous variables and log-transformed, while the other studies used cut-points to distinguish high and low concentration groups. The only study to analyze MMP 7 as a continuous variable and to have a longer follow-up time than our study was much smaller (67 patients) [13]. While an association between MMP 7 and outcomes has been reported across multiple cohorts of patients with IPF, findings are inconsistent. In a study of 57 patients with IPF, MMP 7 was not associated with clinical progression or outcome [15]. A study of 97 patients found an association between elevated MMP 7 levels and mortality and transplant-free survival, but not with disease progression identified by a drop in FVC [12]. Future studies will be important to validate our findings.

While our study has several strengths, including the multicenter cohort and the broad array of MMPs and TIMPs assayed, we acknowledge its limitations. We did not include all MMPs nor TIMP 3. While our assays provide precise quantification of circulating concentrations of MMPs and TIMPs, activity and organ-specific quantification cannot be inferred. Additionally, we did not compare the prognostic value of our tested MMPs and TIMPs against other well-documented markers of progression.

**Fig 4:**
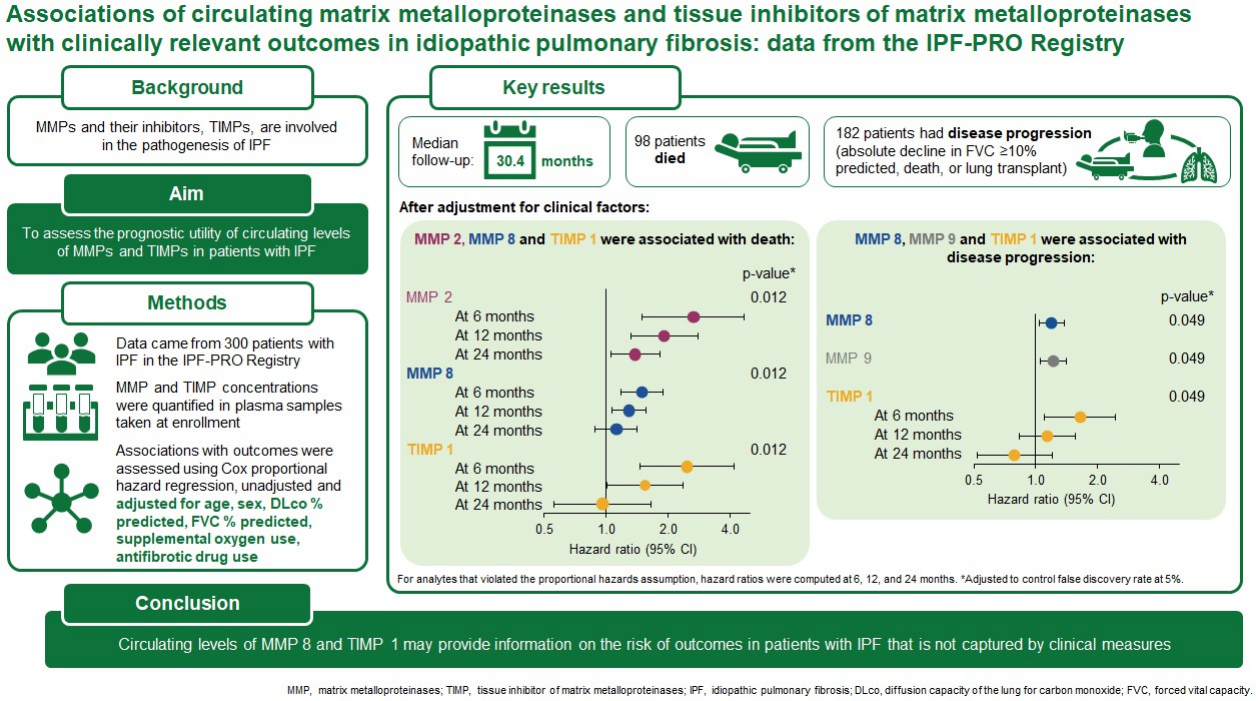
Graphical abstract.

In conclusion, the results of this study further elucidate the potential value of MMPs and TIMPs as disease-related and prognostic biomarkers in patients with IPF. Further validation will be necessary, as well as longitudinal studies. The rich longitudinal data collected in the IPF-PRO Registry, including serial plasma biomarker data, pulmonary function measures, and information on vital status, may yield useful insights to further the goal of improving the diagnosis, prognostication, and management of patients with IPF. A graphical abstract summarizing the findings of this study is provided as Fig 4.

## Supporting information

S1 Methods Methods used to evaluate associations between clinical outcomes and MMP/TIMP ratios of interest based on established biological relationships. (PDF)

S1 Fig Receiver operating characteristic curves. Time-dependent receiver operating characteristic (ROC) curves and area under the curve (AUC) estimates at 6 months (red) and 12 months post-enrollment. Panel A: ROC for MMP2 with time-to-death endpoint. Panel B: ROC for MMP8 with time-to-death endpoint. Panel C: ROC for TIMP1 with time-to-death endpoint. Panel D: ROC for MMP8 with composite time to absolute decline in FVC ≥10%, death, or lung transplant endpoint. Panel E: ROC for MMP9 with composite endpoint. Panel F: ROC for TIMP1 with composite endpoint.
(PDF)

S2 Fig Associations between ratios of circulating MMPs/TIMPs at baseline and death. Hazard ratios per unit increase in baseline log_2_ of each ratio are shown (unadjusted analyses).
(PDF)

S3 Fig Associations between ratios of circulating MMPs/TIMPs at baseline and the composite outcome of an absolute decline in FVC ≥10% predicted, death, or lung transplant. Hazard ratios per unit increase in baseline log_2_ of each ratio are shown (adjusted analyses). Adjustment variables included age, sex, FVC % predicted, DL_CO_ % predicted, supplemental oxygen use at rest or with activity, and antifibrotic medication use, all assessed at enrollment.
(PDF)
