# Supplementary material for: Associations of circulating matrix metalloproteinases and tissue inhibitors of matrix metalloproteinases with clinically relevant outcomes in idiopathic pulmonary fibrosis: Data from the IPF-PRO Registry
Source: PLoS One. 2024 Oct 17;19(10):e0312044. doi: 10.1371/journal.pone.0312044 (PMC11486396; doi:10.1371/journal.pone.0312044)
Supplement: S1 Methods — (PDF) [file pone.0312044.s001.pdf]

Supporting information

S1 Methods. Methods used to evaluate associations between clinical outcomes and MMP/TIMP ratios of interest based on established biological relationships

The ratios of MMP2/TIMP1, MMP8/TIMP1, MMP9/TIMP1, and MMP2+8+9/TIMP1 were derived based on raw concentrations and $\log_2$-transformed. Cox proportional hazards regression was used to assess unadjusted and adjusted associations between each MMP/TIMP ratio and time to death (S1 Fig) and the composite of an absolute decline in FVC ≥10% predicted, death, or lung transplant (S2 Fig). For each outcome, p-values were corrected for multiple comparisons using the Benjamini-Hochberg procedure to control the false discovery rate (FDR) at 5%.
