# Supplementary material for: Associations of circulating matrix metalloproteinases and tissue inhibitors of matrix metalloproteinases with clinically relevant outcomes in idiopathic pulmonary fibrosis: Data from the IPF-PRO Registry
Source: PLoS One. 2024 Oct 17;19(10):e0312044. doi: 10.1371/journal.pone.0312044 (PMC11486396; doi:10.1371/journal.pone.0312044)

Supporting information

S1 Fig. Receiver Operating Characteristic Curves. Time-dependent receiver operating characteristic (ROC) curves and area under the curve (AUC) estimates at 6 months (red) and 12 months post-enrollment. Panel A: ROC for MMP2 with time-to-death endpoint. Panel B: ROC for MMP8 with time-to-death endpoint. Panel C: ROC for TIMP1 with time-to-death endpoint. Panel D: ROC for MMP8 with composite time to absolute decline in FVC ≥10%, death, or lung transplant endpoint. Panel E: ROC for MMP9 with composite endpoint. Panel F: ROC for TIMP1 with composite endpoint.

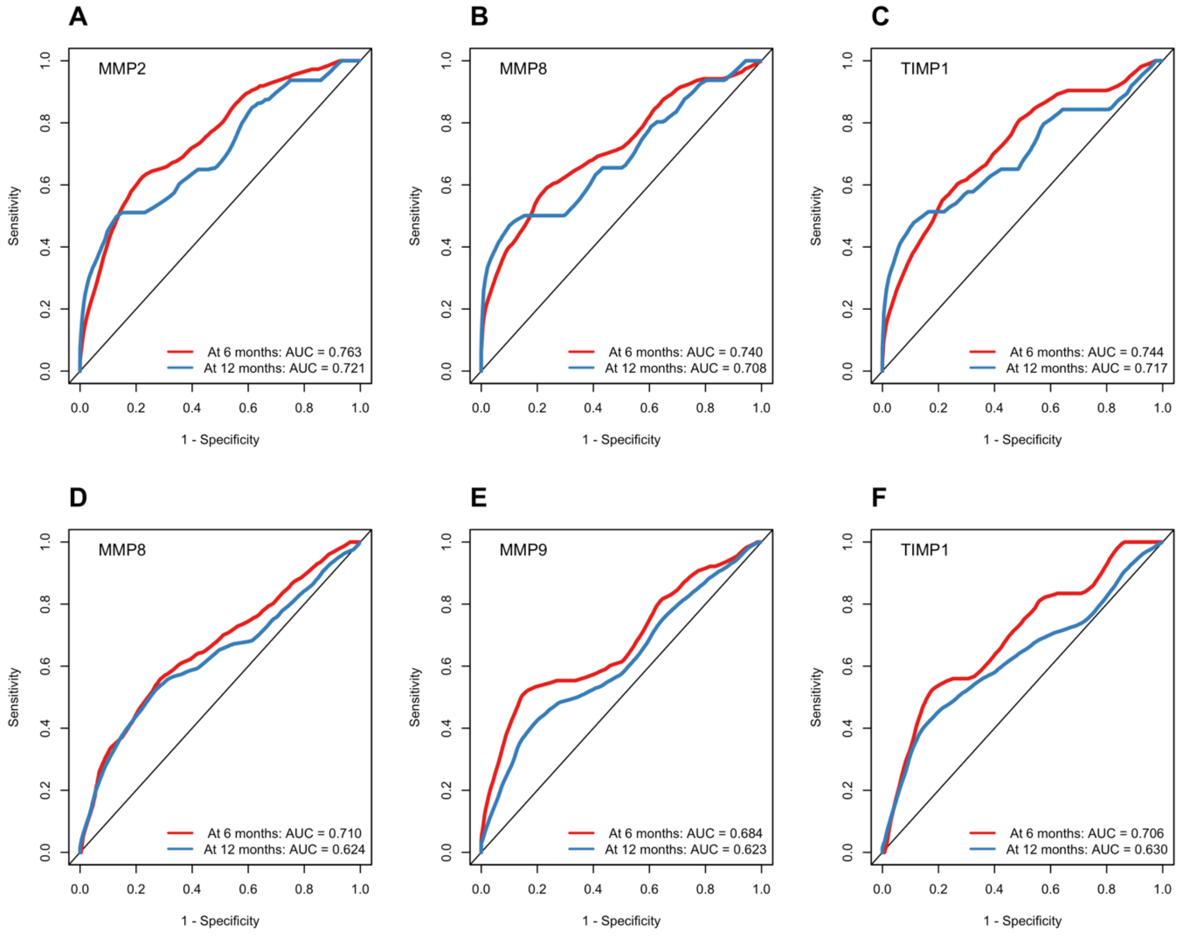

Supplement: S1 Fig — Time-dependent receiver operating characteristic (ROC) curves and area under the curve (AUC) estimates at 6 months (red) and 12 months post-enrollment. Panel A: ROC for MMP2 with time-to-death endpoint. Panel B: ROC for MMP8 with time-to-death endpoint. Panel C: ROC for TIMP1 with time-to-death endpoint. Panel D: ROC for MMP8 with composite time to absolute decline in FVC ≥10%, death, or lung transplant endpoint. Panel E: ROC for MMP9 with composite endpoint. Panel F: ROC for TIMP1 with composite endpoint. (PDF) [file pone.0312044.s002.pdf]
