# Supplementary material for: Associations of circulating matrix metalloproteinases and tissue inhibitors of matrix metalloproteinases with clinically relevant outcomes in idiopathic pulmonary fibrosis: Data from the IPF-PRO Registry
Source: PLoS One. 2024 Oct 17;19(10):e0312044. doi: 10.1371/journal.pone.0312044 (PMC11486396; doi:10.1371/journal.pone.0312044)

Supporting information

S2 Fig. Associations between ratios of circulating MMPs/TIMPs at baseline and death.

Hazard ratios per unit increase in baseline $\log_2$ of each ratio are shown (unadjusted analyses).

	HR (95% CI)	P-value	FDR p-value
**Death**			
(MMP-2+8+9)/TIMP-1	1.28 (0.92, 1.78)	0.139	0.186
MMP-2/TIMP-1	1.16 (0.90, 1.51)	0.249	0.249
MMP-8/TIMP-1	1.16 (1.00, 1.36)	0.054	0.118
MMP-9/TIMP-1	1.18 (0.99, 1.41)	0.059	0.118
**Composite of absolute decline in FVC ≥10% predicted, death, or lung transplant**			
(MMP-2+8+9)/TIMP-1	1.26 (0.99, 1.62)	0.065	0.087
MMP-2/TIMP-1	1.05 (0.90, 1.24)	0.522	0.522
MMP-8/TIMP-1	1.17 (1.04, 1.32)	0.011	0.023
MMP-9/TIMP-1	1.20 (1.06, 1.37)	0.005	0.021

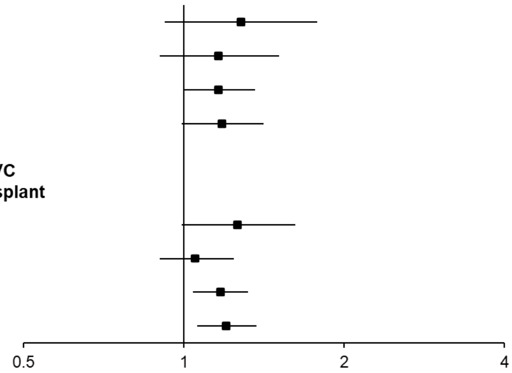

Supplement: S2 Fig — Hazard ratios per unit increase in baseline log2 of each ratio are shown (unadjusted analyses). (PDF) [file pone.0312044.s003.pdf]
