# Supplementary material for: Associations of circulating matrix metalloproteinases and tissue inhibitors of matrix metalloproteinases with clinically relevant outcomes in idiopathic pulmonary fibrosis: Data from the IPF-PRO Registry
Source: PLoS One. 2024 Oct 17;19(10):e0312044. doi: 10.1371/journal.pone.0312044 (PMC11486396; doi:10.1371/journal.pone.0312044)

Supporting information

S3 Fig. Associations between ratios of circulating MMPs/TIMPs at baseline and the composite outcome of an absolute decline in FVC ≥10% predicted, death, or lung transplant. Hazard ratios per unit increase in baseline $\log_2$ of each ratio are shown (adjusted analyses). Adjustment variables included age, sex, FVC % predicted, DL_{CO} % predicted, supplemental oxygen use at rest or with activity, and antifibrotic medication use, all assessed at enrollment.

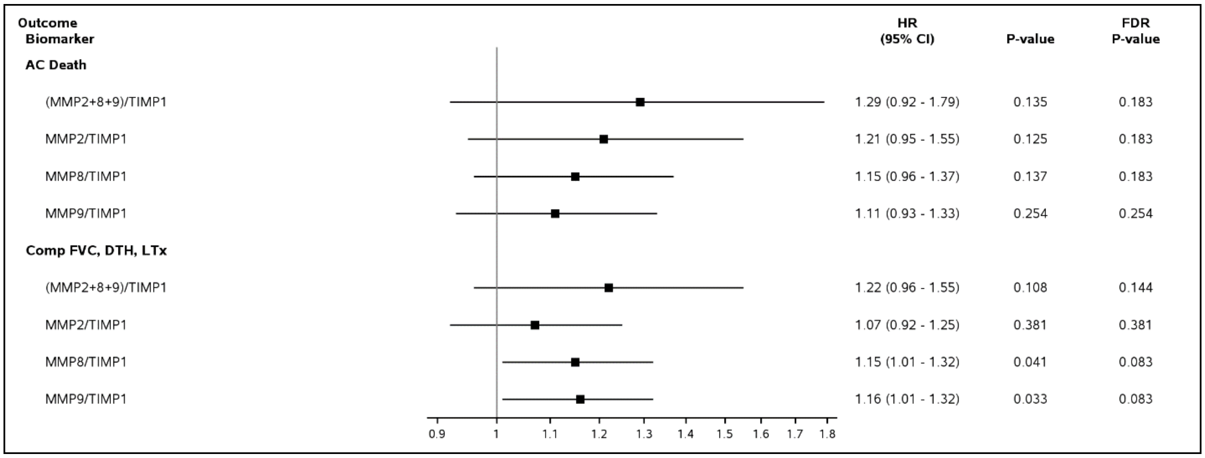

Supplement: S3 Fig — Hazard ratios per unit increase in baseline log2 of each ratio are shown (adjusted analyses). Adjustment variables included age, sex, FVC % predicted, DLCO % predicted, supplemental oxygen use at rest or with activity, and antifibrotic medication use, all assessed at enrollment. (PDF) [file pone.0312044.s004.pdf]
